# The Care of Children’s Teeth

**Published:** 1899-01

**Authors:** 


### THE CARE OF CHILDREN'S TEETH.

Henry S. Belcher, D. D. S., of Buffalo, N. Y., informs us in *The International Dental Journal* that some dentists dread to work on children's teeth. This is a mistake, and we must overcome it, for there is much at stake. This is one of the ways a practice can be built up. One of our most successful practitioners tells me that he secured some of his most influential patients by working for children, gaining the confidence of the little ones by his gentle procedure, and, what is more, the confidence of the parents. We know the importance of preserving the temporary teeth as long as possible, for they nourish the permanent teeth, preserve the shape of the arch, prevent irregularities, etc. A word from the dentist to the child has much more effect than from parents. Tell the child to use a tooth-brush carefully and regularly. Note if they follow your suggestion. If so, remark on their improved appearance, or again speak more decidedly if you notice no improvement. If possible, exclude parents from operating-room. They spoil authority. The child is not to be controlled, but looks to the parent for sympathy, and will not make an effort to control himself. After the dentist has obtained the confidence of the child he can do most anything, so long as he does not deceive. I know of no suitable material with which to fill the roots of temporary teeth. As they must be absorbed, they can not be filled with an insoluble compound. In cases of exposure of the pulp, aristol makes an excellent soothing application. Oil of cloves and tannic acid are also recommended. Dr. Southwick suggests the use of a very minute quantity of arsenic in conjunction with carbolic acid. We have to be satisfied with work that we would be heartily ashamed of in an adult, as it must be done painlessly and quickly. A child will not bear fatigue, and refuses to be hurt. Though the pulp is much less sensitive in a child's tooth than in an adult's, it is much larger in proportion. One-third of a temporary tooth is taken up by the pulp. The pulp lies much nearer the surface, so great care must be taken not to expose it, though we can resort to capping with a greater expectation of success than with an adult. Copper amalgam, if not abused in its use, makes an excellent filling for temporary teeth, the salts of copper having a stimulating effect on tooth-structure and pulp. It is especially valuable in fragile teeth. By some operators copper amalgam has been used indiscriminately and excessively, since which time it has received their condemnation. Notwithstanding this, it is a very valuable agent for the preservation of soft teeth, when used intelligently. Nitrate of silver is a valuable agent to arrest superficial decay of children's teeth. This is especially valuable in early decay; it acts as a check; stimulates the tooth, and encourages secondary deposits. It is used in stick form, and as a solution. Very slight amounts can be used. This agent is rubbed over the decayed part, the tooth being first thoroughly dried. (I dry around it so that the silver will not be distributed through the saliva.) In closing I will quote Dr. Allport: "The great requisite, as has been said, is to gain the child's confidence. I address myself to the child when I make an appointment, and not to the parent. I say, 'When can you come?' thus giving the little patient an impression of a confidential arrangement between himself and me. This often has a favorable effect upon the child, for children appreciate anything that looks like deference to their wishes, and they will usually meet you half-way when you treat them as principals, where they might be difficult of management if you made some mysterious arrangement with the parent, only half understood by them. The secret of handling our little patients successfully is to do but little at any time, avoiding anything like an air of mystery, and always dealing in perfect candor and honesty of intention with them."

